# Morphological Evolution: Bioinspired Methods for Analyzing Bioinspired Robots

**DOI:** 10.3389/frobt.2021.717214

**Published:** 2022-01-14

**Authors:** Eric Aaron, Joshua Hawthorne-Madell, Ken Livingston, John H. Long

**Affiliations:** ^1^ Interdisciplinary Robotics Research Laboratory, Vassar College, Poughkeepsie, NY, United States; ^2^ Department of Computer Science, Colby College, Waterville, ME, United States; ^3^ Department of Cognitive Science, Vassar College, Poughkeepsie, NY, United States; ^4^ Department of Biology, Vassar College, Poughkeepsie, NY, United States

**Keywords:** selection gradients, morphospace, evolution of morphology, development of morphology, evolutionary robotics

## Abstract

To fully understand the evolution of complex morphologies, analyses cannot stop at selection: It is essential to investigate the roles and interactions of multiple processes that drive evolutionary outcomes. The challenges of undertaking such analyses have affected both evolutionary biologists and evolutionary roboticists, with their common interests in complex morphologies. In this paper, we present analytical techniques from evolutionary biology, *selection gradient analysis* and *morphospace walks*, and we demonstrate their applicability to robot morphologies in analyses of three evolutionary mechanisms: randomness (genetic mutation), development (an explicitly implemented genotype-to-phenotype map), and selection. In particular, we applied these analytical techniques to evolved populations of simulated biorobots—embodied robots designed specifically as models of biological systems, for the testing of biological hypotheses—and we present a variety of results, including analyses that do all of the following: illuminate different evolutionary dynamics for different classes of morphological traits; illustrate how the traits targeted by selection can vary based on the likelihood of random genetic mutation; demonstrate that selection on two selected sets of morphological traits only partially explains the variance in fitness in our biorobots; and suggest that biases in developmental processes could partially explain evolutionary dynamics of morphology. When combined, the complementary analytical approaches discussed in this paper can enable insight into evolutionary processes beyond selection and thereby deepen our understanding of the evolution of robotic morphologies.

## 1 Introduction

For evolutionary roboticists, grand challenges target finding original designs, closing the reproduction loop in physical robots, and allowing for open-ended evolution of physical robots in real environments (Eiben, 2014), which echo the grand challenge from organismal biologists to integrate the analysis of physical and biological systems in order to understand complexity (Schwenk et al., 2009). From this perspective, morphology matters for embodied robots in the same ways that it matters for biological organisms: It permits and constrains individual behavior, and it shapes properties of populations that matter for evolution (Hochner, 2013; Cappelle, et al., 2016). The processes that underlie the evolution of complex morphologies are often themselves complex, for both biological organisms and evolved robots; a deep understanding of evolved morphologies requires techniques to analyze the relevant underlying processes—to answer questions about what morphological forms occur over generational time, and how and why they occur.

One standard technique is a straightforward accounting of forms and traits—e.g., recording which morphological forms or traits occur over generational time—but it alone cannot answer all of the relevant questions. In this paper, we describe *selection gradient* analysis and *morphospace walk* techniques from evolutionary biology, to extend roboticists’ analytical toolkit for investigating evolved morphology. To demonstrate these techniques, we investigate the evolutionary dynamics of populations of *biorobots* (Webb, 2001; Long, 2012), i.e., robotic models of biological, organismal systems. The biorobots in this paper (first described in Aaron and Long, 2021; Hawthorne-Madell et al., 2021) have bioinspired genomic foundations that result in bioinspired morphologies, and they are digitally simulated and embodied in the sense that they operate according to physical rules, with fitness determined by performance on a simple locomotion task: distance traveled in an empty, flat, terrestrial environment.

In evolutionary robotics, change in a population’s maximum fitness over time is commonly used as the first evidence for adaptation by selection. In comparison, for biologists, the first evidence of adaptation is often change in specific morphological traits (Harvey and Purvis, 1991; Grant and Grant, 2006). Reflecting the observation that traits are not independent evolutionary entities but are genetically, developmentally, and functionally correlated, multivariate methods were created to regress fitness onto morphological traits, generating in a linear model coefficients that serve as directional selection gradients (Lande and Arnold, 1983). When normalized by variance or mean values of the traits, these selection gradients can indicate which morphological traits are targets of selection in any given population and generation.

As previously shown in Roberts et al. (2014), selection gradients may change dramatically over generational time, suggesting complex evolutionary dynamics even in greatly simplified biorobotic models. We significantly extend that previous work by applying selection gradient techniques to substantially more complicated biorobots and illustrating their use alongside morphospace techniques. Ultimately, the presentation of selection gradient analysis in this paper is intended to serve two purposes: 1) to describe our specific experimental results, which support conclusions similar to those of Roberts et al. (2014); and 2) to illustrate its general applicability to the evolved morphology of robots. Indeed, in Roberts et al. (2014), analysis was on simple systems—with only 3 traits, 10 generations, and populations of size 18 (6 genotypes, with 3 clones of each)—whereas selection gradient analysis in this paper is applied to much more complicated data, requiring additional quantitative tools to handle the large data set, thousands of generations, and complex model selection. Our populations of biorobots demonstrate rich, complex evolutionary dynamics, with different traits becoming most targeted by selection in different generations, but with selection not fully accounting for the resulting morphological change.

Selection, in that sense, is not enough. Driver mechanisms other than selection contribute to evolved morphologies (Roberts et al., 2014), and in this paper we also present and discuss morphospace analysis and morphospace walk (*MW*) techniques as complementary to selection gradient (*SG*) analyses. A *morphospace* is an n-dimensional hypervolume with each dimension defined by a trait selected by the investigator, and it is often used in biology to understand the diversity of evolved body forms (Raup, 1966; McGhee, 2007; Claverie and Wainwright, 2014). We focus on morphospace as a geometric space that differentiates morphologies by their position in that space; for example, with segmented and branching robot bodies, morphologies could be distinguished by how many segments and branches occur in each. With this focus on phenotype rather than fitness or genotype, we can map “evolutionary walks” of populations through a morphospace by tracking means or medians of values (e.g., number of branches or segments in an individual) over generational time; each step in an evolutionary walk corresponds to a generation, reflecting generational changes within the morphospace dimensions. Using *random morphospace walk* (*RMW*) analysis, in which each step in a walk is generated by simple, fully probabilistic processes—in contrast to steps in evolutionary walks, which are based on observed evolutionary data—we compare our evolutionary walks to random walks in the same morphospace, exploring hypotheses about how possible biases in driver mechanisms might be reflected in the evolutionary dynamics of our biorobots. As a methodological note, morphospace analysis as we employ it thus acknowledges the importance of explicitly including development in analysis—morphological development can be a key feature in evolution (Kriegman et al., 2018; Salazar-Ciudad, 2021). In this paper, morphospace analyses serve two purposes: 1) presenting a specific experimental case study suggesting that biases in development can influence and partially explain morphological evolution in cases where selection does not fully account for observed morphological trends; and 2) illustrating in general how morphospace walk techniques can be broadly applied to analyze morphologies of robot populations.

With the added control provided by computational methods, biorobots allow investigations of components of evolutionary systems that biologists often lack *in toto*—e.g., the genome, the rules of development, the rate of mutation, and the precise actions of selection—to illuminate elements that can otherwise remain implicit or extricate effects that can otherwise remain conjoined. Along with that capacity comes a need for caution, however, to avoid the possible experimenter bias that can accompany a detailed knowledge of the internal representations. Because biologists do not have that window into natural, living organisms, the selection gradient and morphospace walk methods in this paper rely only on externally observable traits and properties; this is consistent with some other morphological analysis of evolved robots (e.g., species determination in Medvet et al., 2021), but it reflects a complementary perspective to analytical approaches founded in knowledge of underlying genetic encodings (e.g., Miras, 2021).

Indeed, these techniques—1) straightforward accounting of morphological traits and forms, 2) selection gradient analysis, and 3) morphospace analysis—are fundamentally grounded in observed robotic (or organismal) behavior and morphological traits; taken together, they provide a foundation for illuminating the integrated effects of evolutionary mechanisms underlying evolved morphologies, applicable to robot systems even without experimenter knowledge of the underlying computational models and algorithmic details. To our knowledge, the selection gradient techniques in this paper are substantially more sophisticated than any previous application of selection gradients to robotics, the morphospace walk techniques spotlighted in this paper have not previously been applied to robotics, and biased random morphospace walks are novel to this paper. Below, we describe these techniques in more detail and present results of their application to our populations of biorobots.

## 2 Overview of the Techniques

The three techniques presented in this paper may be used separately or in combination with each other. In this paper, we focus on a case that illustrates their combined usage, but the purposes of each component technique, and their roles in understanding evolutionary dynamics, remain the same whether applied in isolation or combination.

• **Straightforward accounting**: The standard way to record and illuminate what occurs in the evolution of a population. It is included here not for its novelty, but because it is generally useful, and it is used here to provide foundations for other analytical methods. In this paper, its uses include recording fitness values, occurrences of morphological forms, relative prevalence of morphological traits, and how they all vary over generational time.
• **Selection gradient analysis**: Indicates which morphological traits are targeted by selection over generational time, and to what extent they are targeted. Investigators first determine a set of traits to be considered, and analysis is performed with respect to those traits.

The shortcoming of analyzing each trait as if it were independent—which can commonly occur with straightforward accounting (as in our analysis in Section 4.2), but which is not the case in any integrated agent—can be overcome by analyzing all traits at the same time; covariance among traits can be measured, and models of different subsets of variables can be selected and compared. In each population and generation, the direct impact of selection on all traits can be analyzed using multiple linear regression, regressing fitness onto traits of interest, yielding coefficients for each trait, standardized by trait variance or mean (see Hereford et al., 2004). These coefficients, called selection gradients, partition the effect of directional selection acting on behavior into its impact on separate morphological traits considered simultaneously (Lande and Arnold, 1983).

In this paper, we consider sets of *mechanical* and *sensorimotor* traits of our biorobots (see Section 4.2), and selection gradient analysis demonstrates the relative importance to selection of each set, as well as the relative importance of individual components of each set. It further enables the statistical determination of how much of the variance in fitness is explained by each set of traits. The determination that selection does not fully explain variance in fitness motivates the further investigation using morphospace techniques, and the indication of the importance of one particular trait (see below) guides the focus of our attention in our morphospace analysis.

• **Morphospace analysis**: Projects information about the occurrence of morphological forms and traits onto an *n*-dimensional space, to illuminate and focus the effects of morphology along those dimensions. In this paper, with our segmented and branching biorobots that are selected for reproduction based on locomotion performance, we focus on the 2-dimensional morphospace with axes of branches (*b*) and segments (*s*). In this morphospace, we record which forms occurred in the evolution of our biorobots, to begin to understand the limits of evolutionary dynamics along these dimensions. We further represent evolutionary dynamics using *evolutionary walks* in morphospace, to represent how populations varied from generation to generation along these dimensions, and we compare evolutionary walks to *random morphospace walks*, enabling the investigation of how biases, such as those that could be implicit in development, might affect evolutionary dynamics.

As one specific example of the conjoined application of these techniques to the biorobots in this paper: straightforward accounting determines that the change in the branch-to-segment ratio $\frac{b}{s}$ slows after a certain point in generational time; selection gradient analysis indicates that ratio is an especially important target of selection, though much of the variance in fitness of our populations is not explained by selection on mechanical morphological traits; and morphospace analysis shows that a possible developmental bias is consistent with the observed evolutionary change in this ratio over the generations *before* the change in $\frac{b}{s}$ slows but not over generations *after* it slows, suggesting a role of development in explaining the fitness in variance that selection does not.

Foundational to these techniques is the atomization of the robots into component traits, which serve as foundations for analysis (e.g., the axes of morphospace, the variables over which regressions are performed). As expected, different choices would yield different results, but once those key choices are made, these techniques can be applied broadly. For example, we do not in this paper explore differences among encoding schemes (cf. Miras et al., 2018; Veenstra and Glette, 2020; Miras, 2021), environments (cf. Auerbach and Bongard, 2014), morphological descriptors (cf. Miras et al., 2018), diversity metrics (cf. Samuelsen and Glette, 2014), developmental processes (e.g., Joachimczak et al., 2014), or forms of reconfigurable robots (e.g., Kriegman et al., 2018; Medvet et al., 2021; Talamini et al., 2021), but as long as investigators identify traits and values to record, fitness metric and morphospace axes to employ, biases to investigate, etc., these analytical techniques can be applied broadly.

This paper investigates only robots with a particular bioinspired genome and development process, but our example is intended to illustrate broad applicability of these techniques, not to delimit their applicability—investigations into other components, dimensions, or evolutionary dynamics could also be supported by the three approaches highlighted here. In the sections that follow, we describe in more detail the biorobots used in our exploratory study, along with our experiments and analyses that apply these three techniques.

## 3 Methods: Biorobots

The foundations of our work are simulated biorobots with bioinspired genomes, developmental processes, and morphological traits. Below, we briefly describe details of our robots that are most immediately relevant for this paper; for additional details about the robots and our *Embodied Computational Evolution* framework, see Aaron and Long (2021), Hawthorne-Madell et al. (2021).

### 3.1 Biorobots

**FIGURE 1:**
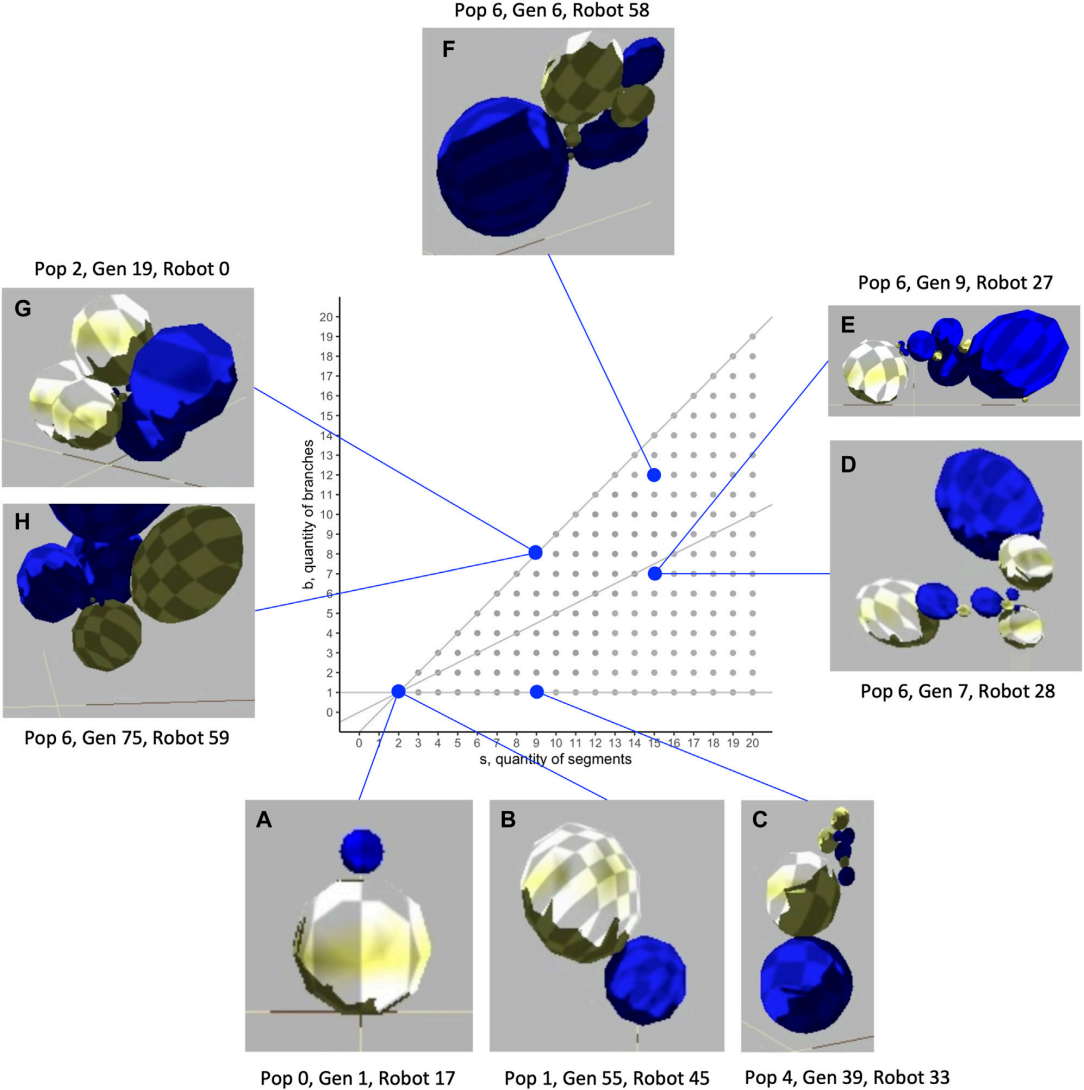
Morphological diversity of evolved biorobots in the segment-branch morphospace. These biorobots were chosen to highlight the range of mechanical morphologies that were evolved and to show the indeterminate relation that sometimes exists between morphology and fitness. While two robots both have two segments and one branch **(A, B)**, Robot A fails to move (lack of a motorized joint; fitness, w=0 ), while Robot B moves by flexing the joint (not shown) between the spheres, rotating, and bouncing away (w=360) . Robot **(C)** is elongated, with a single branch and multiple segments (w=132) . Intermediate in form between elongated and dendroidal body architectures are Robots **(D)** (w=70) and **(E)** (w=11) . Dendroidal forms, with almost as many branches and segments, are shown by Robots **(F)** (w=39) , **(G)** (w=4) , and **(H)** (w=172) . Spherical segments are distorted in some views because of the wide-angle effect of the camera view. Videos of each robot may be found in the supplemental materials. All biorobots were selected from the experimental treatment of 0.0035 mutation rate.

The biorobots are constructed of spherical segments connected by joints to each other, both in series and in parallel branches. The size, number, and relative orientation of segments can vary. In the populations evolved in our study, biorobots had as few as two and as many as 16 segments (Figure 1), with segments arranged in as few as one and as many as 12 branches.

**FIGURE 2:**
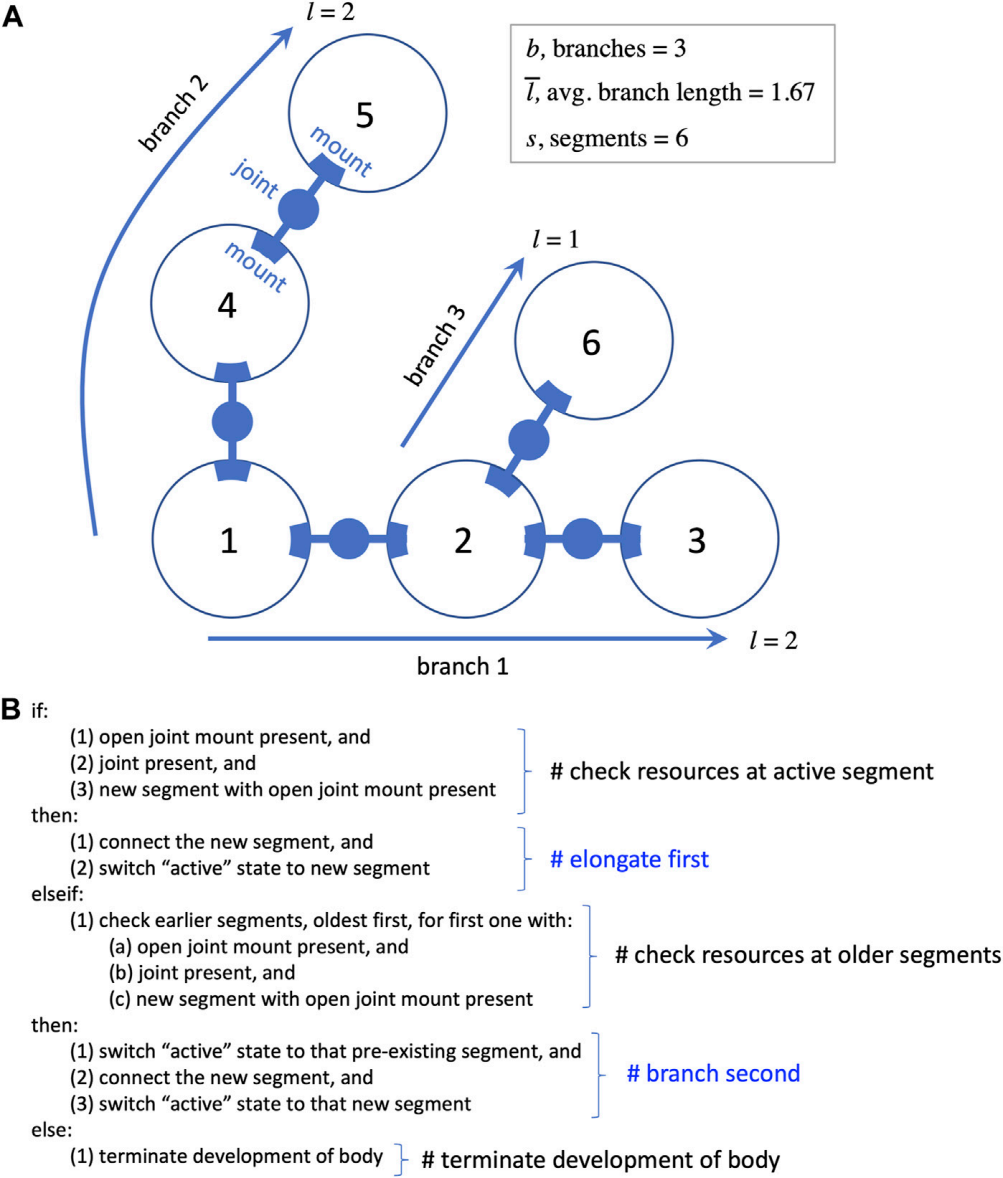
Development of the biorobot’s segmented and branched body. **(A)** Every biorobot is built from 3D spherical segments (represented as circles here) connected by joints that attach to mounts. Sensorimotor morphologies—sensors, wires, and neurons—are not shown. During development, each segment is added to the next in order in which it finished development (numbers in circles). The length of a branch is the segments added to the base segment to form the branch. **(B)** Rules of segment-branch development in pseudocode. A branch elongates (see branch 1) until the newly added segment runs out of resources, such as open joint mounts (segment 3). Then the algorithm checks for resources starting back at segment 1, until a mount and a joint are found to form a new branch. Elongation is favored by occurring before branching in the algorithm. Elongation and branching continue until segments, open mounts, or joints are depleted, at which time development proceeds to add touch sensors, neurons, and wires to each segment in the order of their segmental development (not pictured).

Each segment may have a variable number of external mounts for touch sensors and joints (Figure 2A), as well as a variable number of neurons located internally. Joints are hinges that may be one of three possible kinds: 1) motorized, with a single angular degree of freedom with a given frequency and amplitude; 2) free to move but lacking a motor; or 3) lacking both a motor and the ability to change angle. Touch sensors may be mounted anywhere on the surface of a segment; they are activated when in contact with the ground or another segment. The activation from a touch sensor may be transmitted to a motor via a complete sensorimotor circuit in two ways: 1) a wire connects it directly to the motor; or 2) a wire connects it to a neuron (group) that is connected to the motor. In addition to wires connecting sensors to motors or neurons, wires can connect neurons to each other, and wires are not necessarily perfect conductors of signal—the weight that a wire applies to the signal it carries is determined by regulatory elements in the developmental process (see Hawthorne-Madell et al., 2021, which also contains many other details of the biorobots, beyond the scope needed for this paper). The five types of parts noted here—segments, joints, sensors, wires, neurons—provide the full composition of the robots (see Figure 3 in Hawthorne-Madell et al., 2021).

Locomotion is initiated when touch sensors are activated by a biorobot’s contact with the ground or with itself; a signal is then sent to a motorized joint that reconfigures the body on either side of the joint. This reconfiguration can cause new contact points and stimulate other sensorimotor circuits, thus continuing the process for locomotion.

Each of our biorobots can be described as having two types of morphology: 1) mechanical morphology, with segments and branches that transfer physical momentum; and 2) sensorimotor morphology, with sensors, neurons, wires, and circuits that connect sensors to motors. This dichotomy roughly corresponds to concepts of “external” and “internal” morphology, with a functional distinction that is commonly categorized as “body” and “controller” in robotics (although these “controllers,” as described above, are extremely simple compared to many others in robotics).

### 3.2 Genetics

Each biorobot has a continuous, single-stranded genome of fixed length, 16 kb (kilobases), the size of some RNA viruses (Lynch, 2010). Like the biological genome, the bases are quaternary digits; when contained within a gene, they are read and expressed as triplet codons. A total of 64 different codons are possible, and some are redundant at the third base (for example, 000 and 001 both code for a segment). We give a brief overview of the genetic encoding here, containing only details necessary for the applications and results in this paper; for complete details, see Hawthorne-Madell et al. (2021).

Each gene codes for one of the five types of parts mentioned in Section 3.1: segment, joint, sensor, wire, neuron. Only one part per gene is expressed during development, and other sections of the gene express codons that regulate development by controlling the features of parts (e.g., the type of joint) and the duration of processes that determine properties such as the final sizes of segments and the positions and numbers of mounts for the parts. Each gene is defined by a start and stop codon; since those codons may be altered by mutation, the number and length of genes may evolve. During replication of the genome during reproduction, each base pair has a probability of randomly mutating, ranging from 0 to 0.005 in increments of 0.0005.

### 3.3 Development

Development is the genotype-to-phenotype mapping process, assembling parts into a finished agent. The full developmental process is explained in detail elsewhere (Hawthorne-Madell et al., 2021), along with its relation to the genome; we provide a brief overview here, containing details relevant for the applications and results in this paper.

An algorithm reads the genome and creates a pool of parts according to rules about how to translate genes into the five component types. The parts in the pool are retrieved for assembly in the order in which they completed development. Assembly begins by building the main body, connecting segments with joints; at completion, this process has formed one or more branches (Figure 2). The process then proceeds as a series of steps conditional on available resources of segments, joint mounts on segments, and joints. Segments are added in series, elongating the initial branch when possible, with each newly added segment becoming the active point for the next step; once segments are added, they cannot be moved or destroyed later in the process, a biorealistic constraint that we refer to as “irreversibility” (see Figure 2B). If the active segment lacks an available joint mount in the presence of a new segment and new joint, the process switches from elongation to branching; this is the only context in which a new branch may be formed. Proceeding from the original segment in order of connection, the algorithm looks for an available mount. The first available mount receives the new joint and segment, creating a new branch. This new branch is then elongated until branching is required. Elongation and branching swap in that order until one of these conditions terminates this body building: 1) no unattached segments remain; 2) no joints remain; or 3) no open joint mounts remain. This developmental process may build a wide variety of whole-body morphologies. Extreme instances would be purely dendroidal forms—we use the term *dendroidal* to describe highly branched configurations; purely dendroidal forms are those that branch with every additional segment and never elongate—or purely elongated forms that never branch; intermediate forms are also possible.

## 4 Methods: Experiments and Analysis

In this section, we first describe the evolutionary experiments done with our biorobots (Hawthorne-Madell et al., 2021), which generated the morphological data to be analyzed using straightforward accounting, selection gradient analysis, and morphospace walk techniques. Then, we describe the specific steps taken to apply each analytical technique, illustrating the general applicability of the techniques with their application to our study.

### 4.1 Evolutionary Experiments

Nine randomly generated populations of 60 biorobots were created; each population serves as an independent replicate in a statistical sense. Each population was subjected to 99 rounds of selection, mutation, and reproduction (including development), creating a total of 100 generations in a given run. Each population was tested at 11 different rates of mutation, starting from the exact same configuration each time in generation 0, creating a total of 66,000 individuals per population, for 594,000 overall.

Each individual was digitally instantiated in a physics engine (https://bulletphysics.org; v2.82) simulating an empty, flat, terrestrial world. (Additional details about the computing environment, which might be needed for strict replicability, are available upon request.) In each selection trial, each individual was given 501 timesteps of uniform duration (i.e., 501 iterations in a simulation loop) in which to move; each robot starts with its lowest point 0.1 units above the ground (to avoid “cheating” that can occur in simulations with physics engines; see Lehman et al., 2020), and the resulting small drop to the ground initiates its motion. The fitness metric was the distance moved: the linear distance from its starting point to wherever it ended up, measured in the horizontal plane.

The fitness values were used to apply truncation selection to populations. The 30 individuals with the highest fitness values—i.e., greatest distances locomoted—reproduced asexually, with offspring replacing the parents in the succeeding generation: the three highest-ranked robots each made four offspring, robots ranked 4–9 each made three offspring, robots ranked 10–18 each made two offspring, and robots ranked 19–30 each made one offspring; these offspring fully comprise a new population of 60 individuals, maintaining the same population size as in previous generations. Mutation was applied at one of the 11 rates during replication of the parent genome. Other than mutation, there was no randomness in reproduction or development, so a mutation rate of 0 meant that parents cloned themselves, reducing population variation with each generation.

### 4.2 Straightforward Accounting: Fitness and Separate Traits

Among the typical ways to understand how populations respond to selection is to measure changes in fitness over generational time. It is also useful to separately record and examine how each morphological trait changes over generational time, to construct a first approximation of which traits correlate positively or negatively with fitness.

In the experiments described in this paper, we measured the fitness of each of our biorobots by a behavioral measure, distance locomoted. At each level of mutation, locally weighted regressions (LOESS curves, from *geom_smooth*, R v.4.0.3, with span of 0.75, window of 80 points, and y∼ x ) describe the smoothed mean and 95% confidence interval of populations’ mean fitness over time; LOESS is a non-parametric polynomial regression method that fits models robustly to a running window of the data (Irizarry, 2019). This standard analysis can detect and visualize differences between experimental conditions. Complementary information is provided by analyzing minimum, median, and maximum fitness values of populations, which can differentiate population-level evolutionary responses; median fitness is an especially important factor in the example in this paper, because truncation selects the highest half of the population to reproduce.

We also recorded the quantities of 12 morphological traits that occurred in our biorobots. Of the 12 traits, 4 were mechanical morphological traits (Figure 2A): quantity of segments s ; quantity of branches b ; ratio of branches per segment $\frac{b}{s}$ ; and average branch length*,* where the length of a branch is the number of segments added to the base segment to form the branch, as shown in Figure 2. (Symbols are introduced here only for traits that later appear in our morphospace analysis.) The other 8 traits considered were sensorimotor morphological traits: quantity of sensors, quantity of wires, quantity of neurons, quantity of circuits, and then the ratios of those four traits per segment. For the analyses presented here, each trait was pooled across populations, separated by level of mutation, and plotted over generational time.

In general—in our analyses and elsewhere—when an agent is atomized into components for an analysis of this type, an implicit assumption is that those components may evolve independently from each other. That assumption ignores the integrated nature of the locomotor behavior that is the basis of fitness for our biorobots, but nonetheless, the patterns of separate trait evolution are important correlates to help begin to understand how the selection of individuals impacts the evolution of populations.

### 4.3 Selection Gradient Analysis: Targets of Selection

To apply selection gradient (SG) analysis to our biorobot populations, our first round of modeling addressed which morphological traits are targets of selection acting on behavior, and whether those targets change over generational time or with different mutation rates. In each population, generation, and level of mutation, individual fitness was regressed against the 12 morphological traits described in Section 4.2 above: 4 mechanical morphological traits, and 8 sensorimotor morphological traits. Stepwise linear regression with Akaike Information Criterion (*AIC*) for model selection was run (R v.4.0.3) on 9,000 populations (9 replicates, 10 mutation rates, 100 generations); the mutation rate of 0 was not run since clones eliminate the variance needed for regression. The stepwise regression (mixed forward and backward) with AIC was configured to compare all possible models of the set of traits; the best model was the subset with the lowest AIC value, a balance between the goodness of fit and the simplicity of the model. The best model for each population, generation, and mutation level provided the raw regression coefficients of the selected traits; coefficients *β* were calculated by normalizing the raw coefficients by the mean of the traits (Hereford et al., 2004).

Our second round of selection gradient modeling focused on the types of morphological traits identified above—the mechanical and the sensorimotor—and addressed a different question: Which type explains more of the variance in individual fitness? In each population, generation, and level of mutation, individual fitness was regressed separately against two sets of morphological traits, mechanical (4 traits) and sensorimotor (8 traits). Multivariate models were compared using coefficient of determination $R^{2}$ , to compare the explanatory value of the full mechanical and sensorimotor models, without distinguishing the contribution of each component variable to the significance of the model (as was done in the first round of SG analysis).

### 4.4 Morphospace Walks: Randomness, Development, and Evolution

**FIGURE 3:**
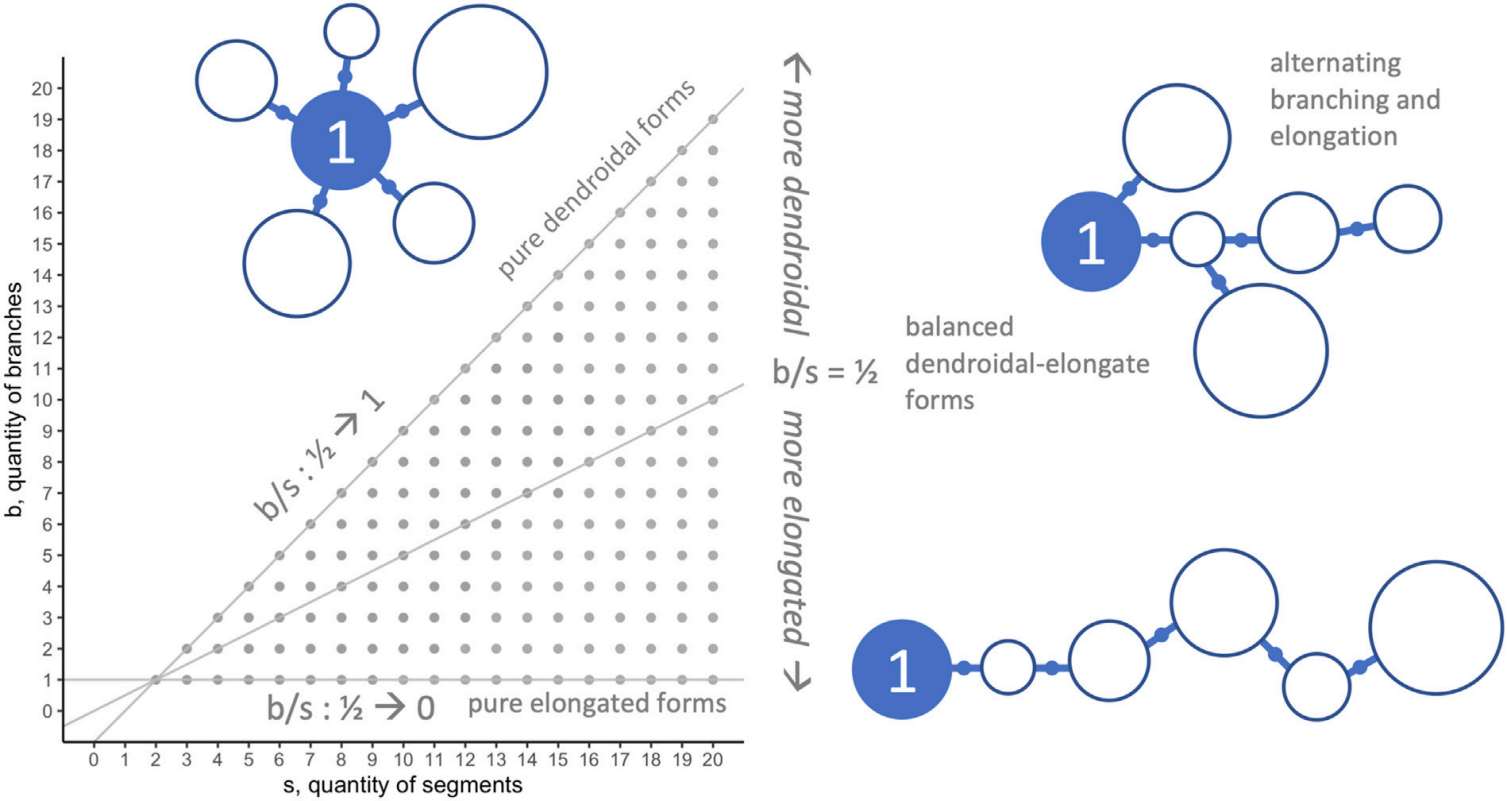
Morphospace: limits and morphological configurations. Because they represent important aspects of the overall morphological configuration (see Figure 1) and development (see Figure 2) of the biorobots, the quantity of segments s and branches b were chosen as the dimensions in this 2D morphospace. This morphospace has limits imposed by development. The upper boundary (gray diagonal) is determined by the developmental rule that one branch has a minimum of two segments, hence s>b . The lower boundary (horizontal gray line at b=1 ) is determined by the developmental rule that elongation may proceed on a single branch until resources are depleted and the agent’s development is complete. Note that the right-hand side of the morphospace has no limit as figured here, with forms of s>20 and b>20 possible.

In general, when applying morphospace techniques, investigators must choose the morphospace axes—the dimensions along which populations will be analyzed (McGhee, 2007). For the morphospace analysis and morphospace walk (MW) techniques in this paper, we analyze our biorobots’ evolved morphologies on two dimensions (Figure 3): number of segments in individual robots (s) ; and number of branches in individual robots (b) . These dimensions were chosen because they permit insights into morphology at the level of whole-body architecture, including elongated or dendroidal forms (see Section 3.3), and being mechanical morphological traits, they are thought to be tightly connected to the locomotor performance targeted by selection. Although straightforward accounting can show what forms occur at different points in generational time or under different experimental conditions (here, mutation rate), MW techniques enable explorations of the roles and comparative impacts of randomness, developmental bias, or selection on the resulting forms.

In our analyses, we use MW models and *randomized morphospace walk* (RMW) techniques to serve three purposes: 1) to show the adaptive trajectory of evolution through the s‐b morphospace; 2) to contrast random walks with the evolved movement of populations in the s‐b morphospace, to help illustrate the directional effects of selection by comparison to an unbiased random trajectory; and 3) to compare biased random walks with unbiased random walks and the evolved movement of populations in the s‐b morphospace, to investigate the effects of a possible developmental bias (the *irreversibility bias*, see below).

To further connect developmental trajectories of individuals—based either on hypothesized developmental biases or on observed evolutionary data—to the possible evolutionary moves of a population in morphospace, we make a number of simplifying assumptions. First, we note that in our system, evolution proceeds by asexual reproduction. If an asexual parent produces an offspring with an identical genome, then that offspring would occupy the same position in the morphospace. Thus, offspring inherit their developmental trajectory in (s‐b) morphospace from their parents, and moves over generational time are constrained by what is possible developmentally. With this foundation for relating development to evolution, we can build an RMW model in which a single point represents either an individual during development or a median value of a population. Note that our simple RMW models are substantial abstractions of the underlying evolutionary processes, lacking any explicit reference to the genome or developmental rules.

The s‐b morphospace (Figure 3) is asymmetric, due to two limits beyond which body forms are impossible. The lower limit is b=1 , since by design any working robot must have at least one branch connecting two segments; this limit represents an extreme of elongated forms. The upper limit is b=s−1 , representing the constraint that the robot must have at least one branch less than the number of segments; this limit represents an extreme of dendroidal forms. The two limits intersect at (2,1) , which represents the simplest possible robot. Note that within these boundaries, there is no upper limit to s or b .

In general, the details of RMW models are determined by experimenters, including boundary conditions, starting points of walks, and what steps are permitted in a walk. (In the text that follows, we let db stand for the change in b and ds stand for the change in s .) In this paper, for simplicity, we restrict db and ds to be −1, 0, or 1 in each step; we handle boundary conditions by treating a call for a robot to move outside of morphospace boundaries as a null step in the relevant dimension(s)—the robot maintains its current value of s or b (or both) until the next step in the walk**.** In total, we consider five different RMWs in our experiments, described here:

• **Fully random (unbiased) walk**: At each step, there are nine equiprobable moves— db and ds are −1, 0, or 1, computed independently.
• **Irreversibility bias (*s*-first) walk**: Intended to represent a potential developmental bias that if development leads to an individual with (s,b) segments and branches, continuing that developmental trajectory could not add a branch without also adding a segment—i.e., the previous configuration of branches cannot be reconfigured later in development. The reasoning stems from the fact that an asexually reproduced offspring must develop on the same pathway as the parent (same numbers of s and b ), and ds=1 represents extending that pathway, possibly (but not necessarily) enabling a new branch, while ds=−1 indicates truncating that pathway, possibly (but not necessarily) preventing a branch from forming; similarly, a change in b is forbidden when ds=0 . So, a step in this walk proceeds by first determining whether ds is −1, 0, or 1 (equiprobable); if ds≠0 , we model as equiprobable whether db=0 or db=ds . This results in five possible (ds,db) steps— (0,0),(1,0),(1,1),(−1,0,), or (−1,−1) —which may in principle be altered by boundary conditions as described above.
• b **-first walk**: Similar to the irreversibility bias walk except with the roles of s and b inverted, representing the possible developmental bias that developmental pathways only result in adding segments when branches are added. (We investigators felt this was less developmentally plausible than the irreversibility bias, but we included it as part of the exploration in this exploratory study.) A step in this walk proceeds by first determining whether db is −1, 0, or 1 (equiprobable); if db≠0 , we model as equiprobable whether ds=0 or ds=db . This results in five possible (ds,db) steps— (0,0),(0,1),(1,1),(0,−1), or (−1,−1) — which may in principle be altered by boundary conditions as described above.
• s **-first, no upper boundary walk**: To explore the role of constraints imposed by the b=s−1 boundary, we also ran the irreversibility bias walk without restricting b to be at most s−1 . Other than this change in the effect of boundary conditions, the walk is identical to the irreversibility bias walk.
• b **-first, no upper boundary walk**: Similarly, we ran the b -first walk without restricting b to be at most s−1 . Other than this change in the effect of boundary conditions, the walk is identical to the b -first walk.

All of our RMW analyses were run in R, with all walks started at the same position, (s,b)=(5,3) , the grand mean of the starting values for all nine populations of biorobots. Each step in a random walk is represented by a single (s,b) value; in the comparative evolutionary walks in our experiments, each step showed the median of a population of 60. To enable comparisons, the same random seed was used for an unbiased walk as for a biased walk to which it was compared. To enable comparison to evolutionary walks, both biased and unbiased RMWs were run for the same number of steps as there were generations of evolution; the final positions, at the last generation or final iteration, were compared. Dimensions b and s , and their ratio $\frac{b}{s}$ , were each analyzed separately with a Kruskal-Wallis test, followed by a Wilcoxon’s test for each pairwise comparison, with Bonferroni correction for multiple test, alpha level 0.05.

Biased RMW models can help provide insight into the impact of biases on evolution, isolated from other possible biases. By comparing data from these biased walks to other walks, we can explore impacts of these biases in contrast to a null model (an unbiased walk) and experimentally observed evolutionary dynamics (the evolutionary walks).

## 5 Results

The evolution of nine populations of 60 individuals under 11 different mutation rates over 100 generations yielded 594,000 individuals of variable morphologies and fitnesses (see Figure 1 and Supplementary Videos). The resulting evolution of morphology is varied and complex. Unless otherwise indicated, all lines plotted in figures are LOESS curves, representing the local conditional mean, with a 95% confidence interval envelope.

### 5.1 Straightforward Accounting: Fitness and Separate Traits

**FIGURE 4:**
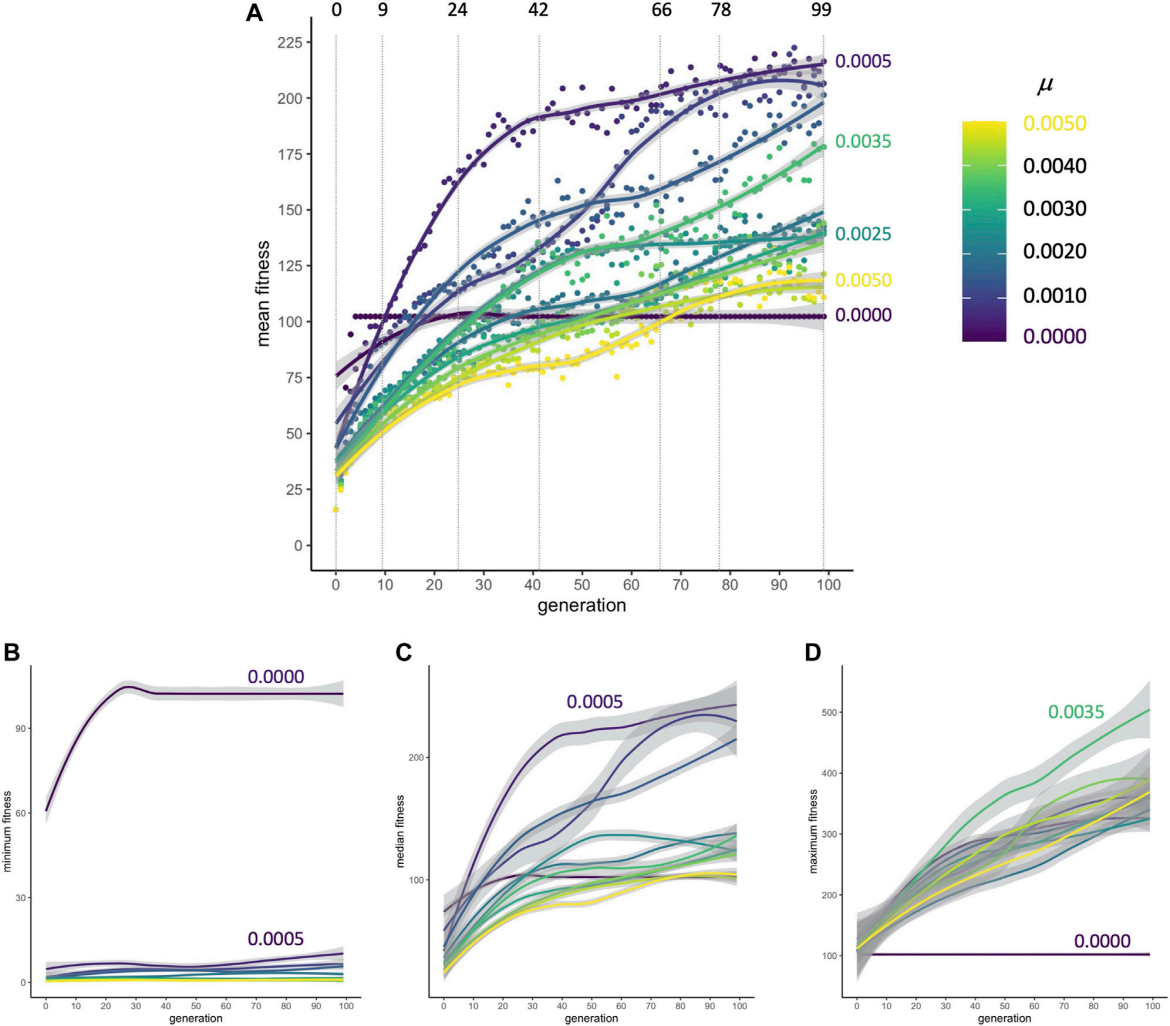
Adaptive evolution. **(A)** Mean fitness. No matter the rate of mutation, populations increase their mean fitness rapidly from generation 0 to 9 in a primary burst. The dashed vertical lines indicate transitional and representative generations that will be sampled in detail to examine the influences of development and selection. Points are means, pooled across populations ( n = 9). Lines are fourth-order polynomial fits, with gray shading representing the local 95% confidence interval. **(B)** The minimum individual fitness is low in every generation except when mutation is absent (violet line), indicating that mutation in asexual lineages disrupts the genomes of at least some of the adapted morphologies. **(C)** Median individual fitness marks the point of truncation for the selection algorithm. **(D)** In contrast to the populations’ mean **(A)**, minimum **(B)**, and median **(C)**, the maximal fitness of individuals increases steadily when mutation is present. For **(B–D)**, the lines are locally-weighted regressions (LOESS) representing the mean of that measure, pooled across populations, with gray shading representing the local 95% confidence interval ( n = 540).

When populations are subjected to selection for improved locomotion, mean fitnesses rapidly increase from their randomly generated starting conditions at generation 0 to generation nine; this occurs for every rate of mutation *μ* (Figure 4A). From generations 9 to 42, the rate of change in fitness drops. By generations 72 to 99, fitness in populations in some of the *μ* conditions has plateaued. Because populations without mutation evolve by simple cloning, the minimum fitness of individuals in those populations is higher compared to populations where novel variants are being generated in each reproductive cycle; the presence of individuals at or near zero fitness in every generation shows that mutation can disrupt adapted but brittle asexual genomes (Figure 4B). In spite of these genomic disruptions, as was the case with mean fitness, the populations’ median fitness increases and plateaus (Figure 4C). With mutation generating variation, the maximum fitness of individuals increases at a linear pace for all 100 generations (Figure 4D). Each of the nine populations undergoes evolution under 11 different rates of mutation from 0 to 0.005 in 0.0005 increments; note that the condition for the best overall population fitness (*μ* = 0.0005, see Figures 4A,C) does not produce the best individuals (*μ* = 0.0035, in 4D).

**FIGURE 5:**
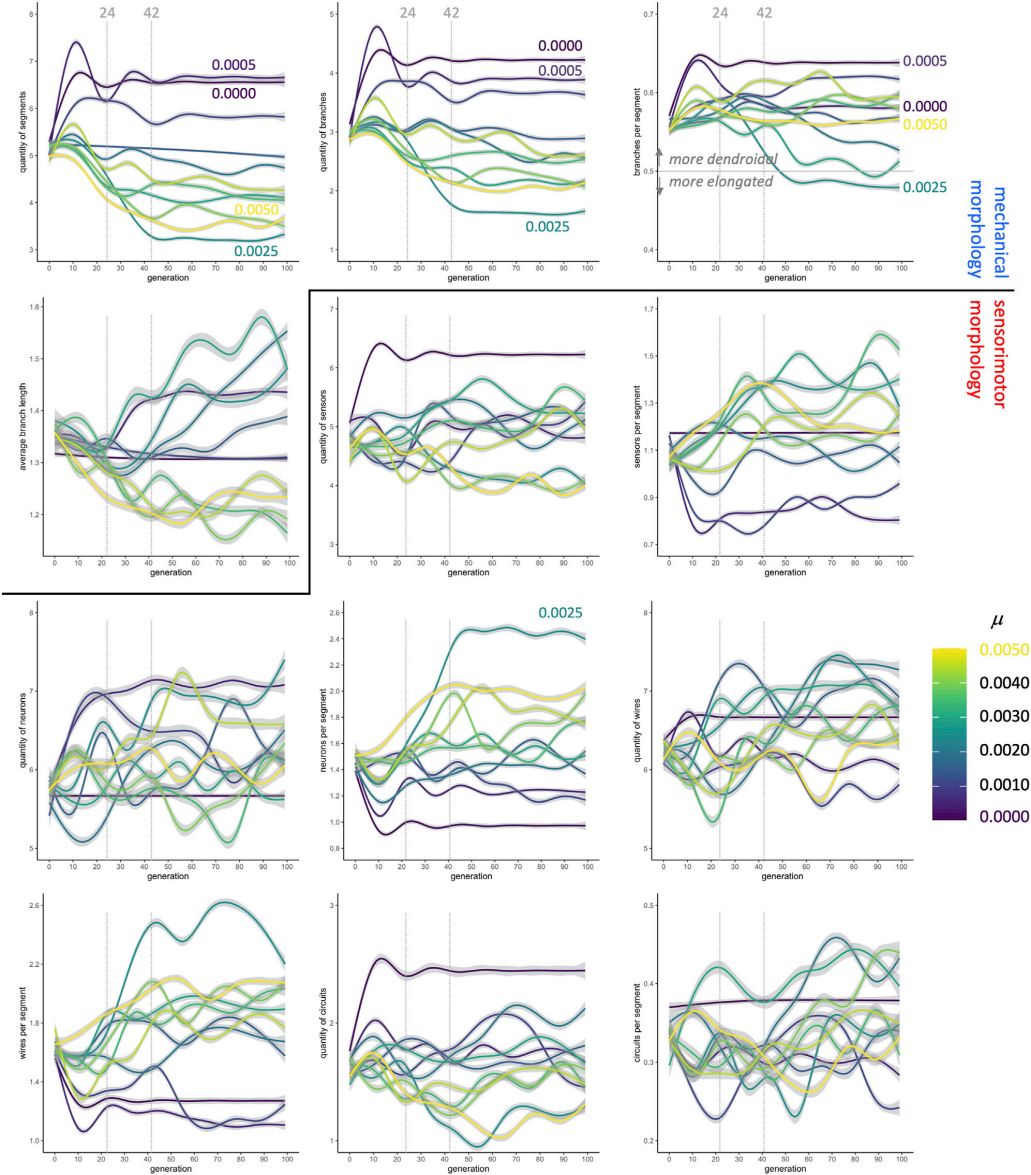
Evolution of morphology as separate traits. The quantity of segments, the quantity of branches, and the ratio of branches to segments **(top row)** change dramatically in the first 24 generations, stabilizing in most conditions by generation 42. In contrast, the sensorimotor morphologies, beginning with the neurons, are more variable over time and between conditions. Lines are means with gray 95% confidence intervals.

Fitness, however, does not immediately reveal the entire morphological story: When morphological traits are each analyzed independently, the patterns (Figure 5) do not map consistently onto fitness patterns (Figure 4). Consider, for example, the changes in values of traits over the first 24 generations, which show variation across different rates of mutation. There is a consistent pattern of increasing values at low *μ* and decreasing values at high *μ* among the s , b , and $\frac{b}{s}$ traits of mechanical morphologies (top row, Figure 5); by comparison, the patterns are highly variable for the traits of sensorimotor morphologies over both mutation rate and generation. In addition, there is no apparent correlation between these morphological trait patterns and the fitness patterns (Figure 4) over the first 24 generations. This variation across traits and experimental conditions is intriguing, and it shows the challenge of interpreting the evolution of morphology piece by piece—and, thus, the importance of methods for addressing this challenge, such as selection gradient analysis.

### 5.2 Selection Gradients: Morphological Targets

**FIGURE 6:**
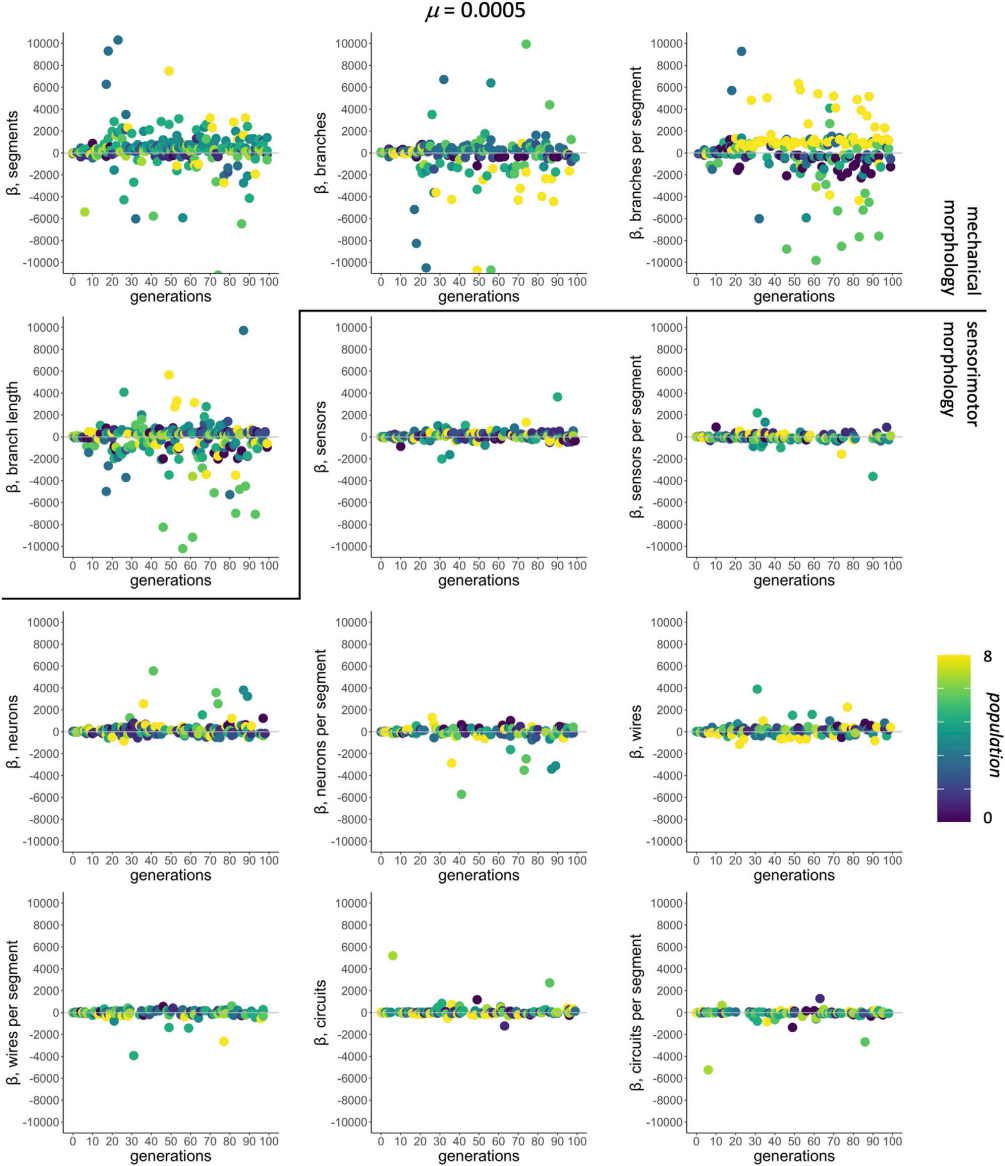
Selection gradient analysis: morphological targets. The subset of significant mean-standardized directional selection gradients chosen by AIC from all 12 traits are shown by population (different shades). The mutation condition μ=0.0005 is shown here because it evolves the populations with the highest mean fitness (see Figure 4); we see similar trends at other non-zero levels of mutation.

In the context of selection and its targets, correlations among traits are assessed by multivariate models that regress individual fitness onto all 12 morphological traits simultaneously. For *μ* of 0.0005, selected as an example because it was the rate that produced populations with the greatest mean fitness (see Figure 4), the mean-standardized selection gradients *β* have larger magnitudes, positive or negative, for the traits of mechanical morphology than for those of sensorimotor morphology (Figure 6).

**FIGURE 7:**
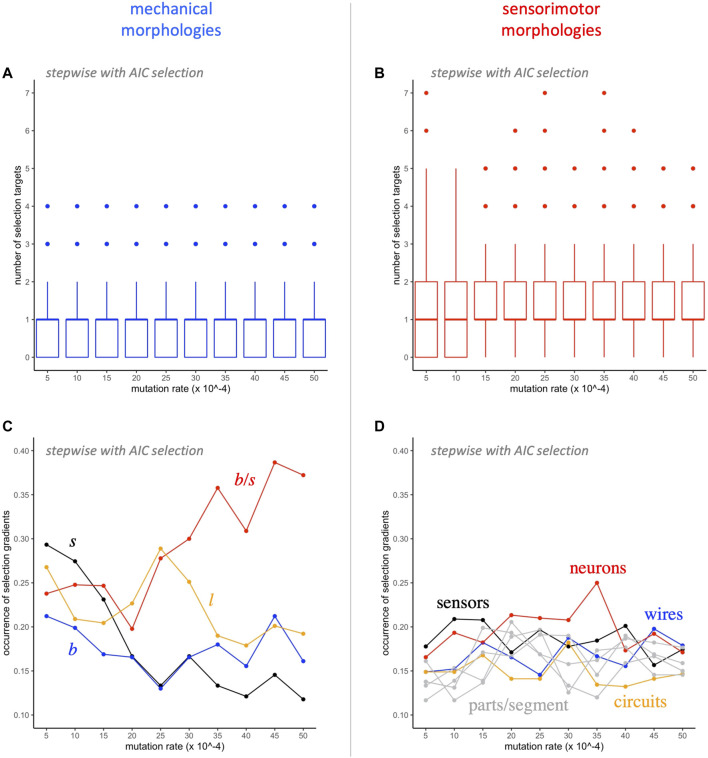
Selection gradient analysis: switching morphological targets. A median of one mechanical **(A)** and one sensorimotor **(B)** morphology is targeted by selection at each level of mutation. The occurrence of the different mechanical morphological traits **(C)** diverges as the rate of mutation increases, with $\frac{b}{s}$ increasing to nearly 40% while the occurrence of the other traits decreases. This pattern is absent in the occurrence of the different sensorimotor traits. Each mutation rate contains 900 models **(A, B)**, with a single point summarizing the rate of occurrence for a given trait **(C, D)**.

Further analysis of the traits targeted by selection shows a number of interesting patterns (Figure 7). Even though there are only 4 mechanical traits, compared to 8 sensorimotor traits, a median of one of each type is represented in each model, independent of the level of mutation (Figures 7A,B). Note that the distributions of the two types differ: In all but the two lowest levels of mutation, mechanical traits have interquartile range between 0 and 1, and sensorimotor traits have interquartile range between 1 and 2. Each of the 12 traits occurs in at least 10% of the models (Figures 7C,D). As levels of mutation increase, the occurrence of the ratio of branches to segments, $\frac{b}{s}$ , increases dramatically to nearly 40% of the models, even as other mechanical morphologies decrease and as sensorimotor morphologies are relatively stable. This indicates that $\frac{b}{s}$ is the trait most often targeted by selection.

**FIGURE 8:**
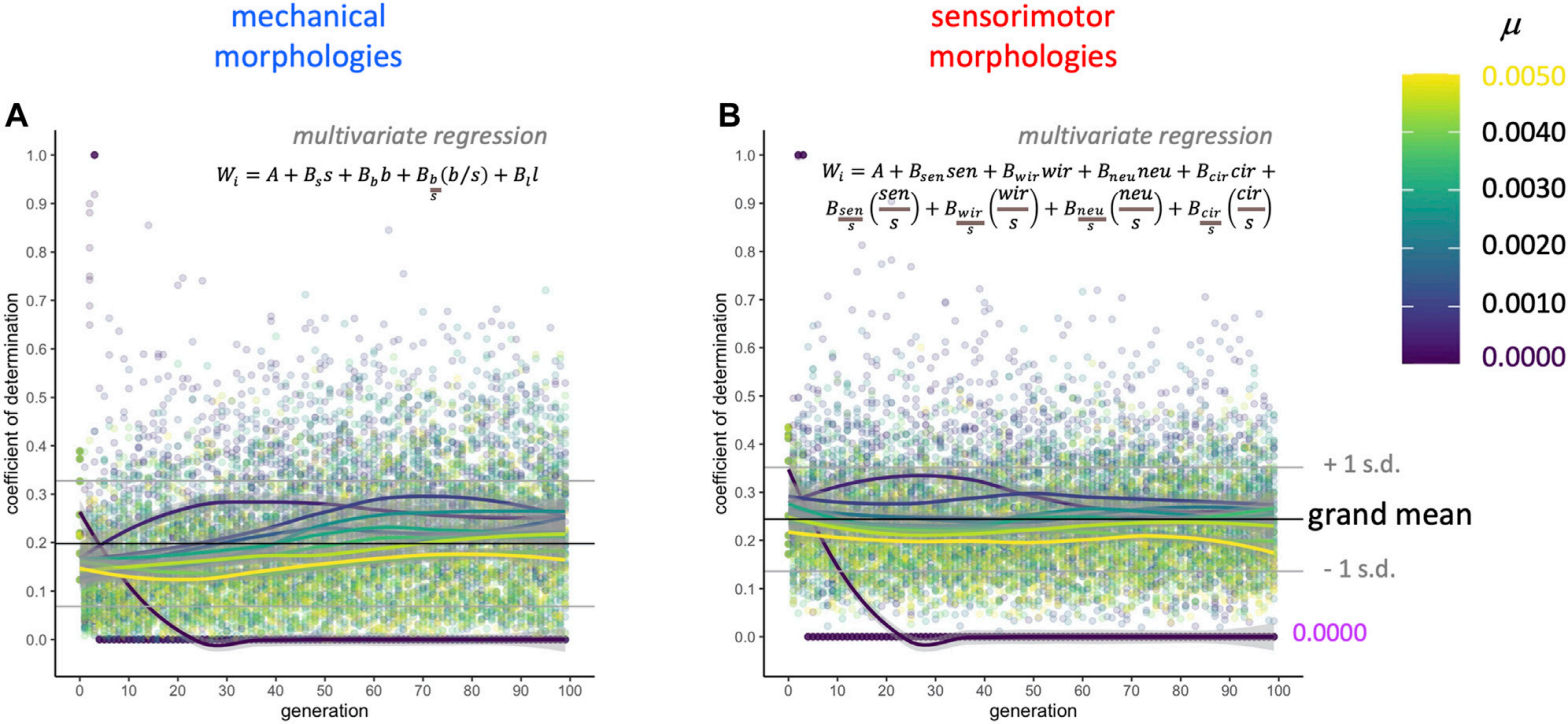
Selection gradient analysis: morphology explains only part of the variance in fitness. Two multivariate regression models were run on each population (see equations, above) at each level of mutation and each generation: **(A)** the full set of four mechanical morphologies and **(B)** the full set of the eight sensorimotor variables. The four mechanical morphologies explain about 20% (grand mean) of the overall variance in fitness as measured by the coefficient of determination. The eight sensorimotor morphologies explain about 24% (grand mean) of the variance in fitness as measured by the coefficient of determination.

Complementary to the AIC-stepwise approach, in which different subsets of variables are selected in each model, linear regression models that force inclusion of the full mechanical and sensorimotor data sets represent the upper limit for morphology explaining the variance in fitness in a linear model (Figure 8). Keeping in mind that these two sets of models are not statistically independent, since they are part of the same data set, the coefficients of determination ( $R^{2}$ value) show that these sets of mechanical and sensorimotor traits explain a mean of 20 and 24% of the variance in fitness, respectively. Note also that analyzing different sets of traits (e.g., the descriptors in Miras et al., 2018) could of course yield different results, but our traits enable analysis regarding all of the kinds of parts coded by our robots’ genomes (see Section 3.1), as well as a measure of how relatively elongated or highly branched a form is $\left(\frac{b}{s}\right)$ and other ratios to normalize quantities by the number of body segments in a morphological form.

This selection gradient analysis thus demonstrates that directional selection on these traits only accounts for a portion of the change in fitness over generational time. Some of the remaining variance may be correlated with non-linear (i.e., non-directional) selection, either disruptive or stabilizing selection (Blows and Brooks, 2003). These nonlinear selection effects are detected with quadratic terms, and are of small effect, relative to directional selection, in natural systems (Kingsolver et al., 2001). Unlike when studying natural systems, with biorobotic evolution we know the selection pressure; since it was directional (“linear”) here, we only looked for those effects. Also, this decision to use linear gradients minimizes concerns about over-fitting and computational problems with least-squares models when there are correlations among variables.

### 5.3 Morphospace Analysis

Selection gradient analysis demonstrates that selection does not fully account for the observed variance in fitness. Morphospace analysis suggests that development may also be a factor in this variance.

**FIGURE 9:**
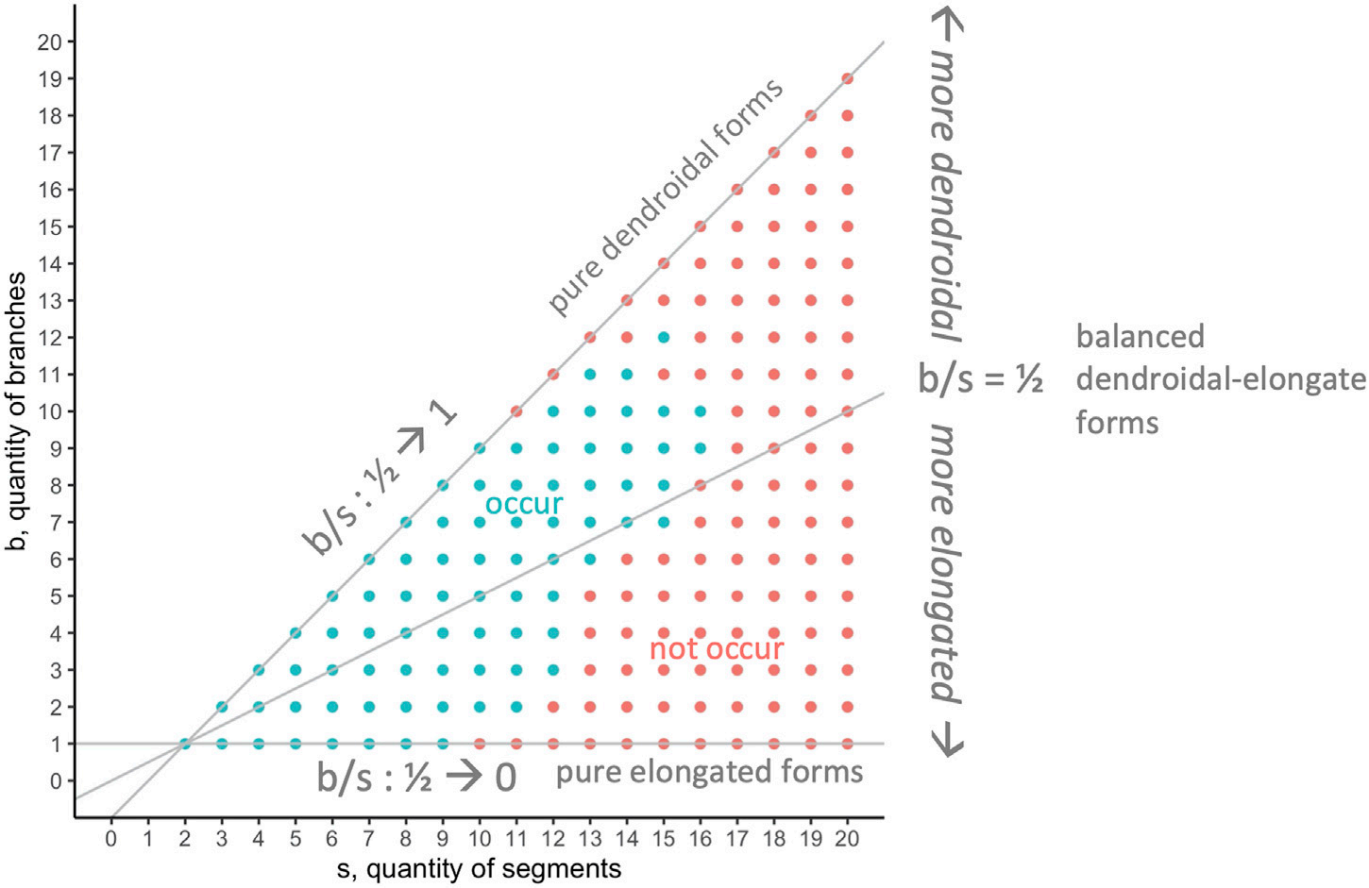
Morphospace analysis: occurrence of s‐b body forms. Of all possible body forms, only a portion of the possible forms (aqua) evolved in 9 populations, 100 generations, and 11 different conditions of mutation (594,000 individuals).

The 594,000 evolved individuals occupy 190 positions in the segment-branch s‐b mechanical morphospace; no forms evolved with more than 16 segments or 12 branches (Figure 9). Body configurations, as measured by the ratio $\frac{b}{s}$ , varied from forms that approach a purely elongated shape ( $\frac{b}{s}=0.111$ , minimum) to those that approach a purely dendroidal form ( $\frac{b}{s}=0.889$ , maximum). Note that most individuals with more than 12 segments are in the region of morphospace above the $\frac{b}{s}=\frac{1}{2},$ line, indicating morphological forms more dendroidal than elongated.

**FIGURE 10:**
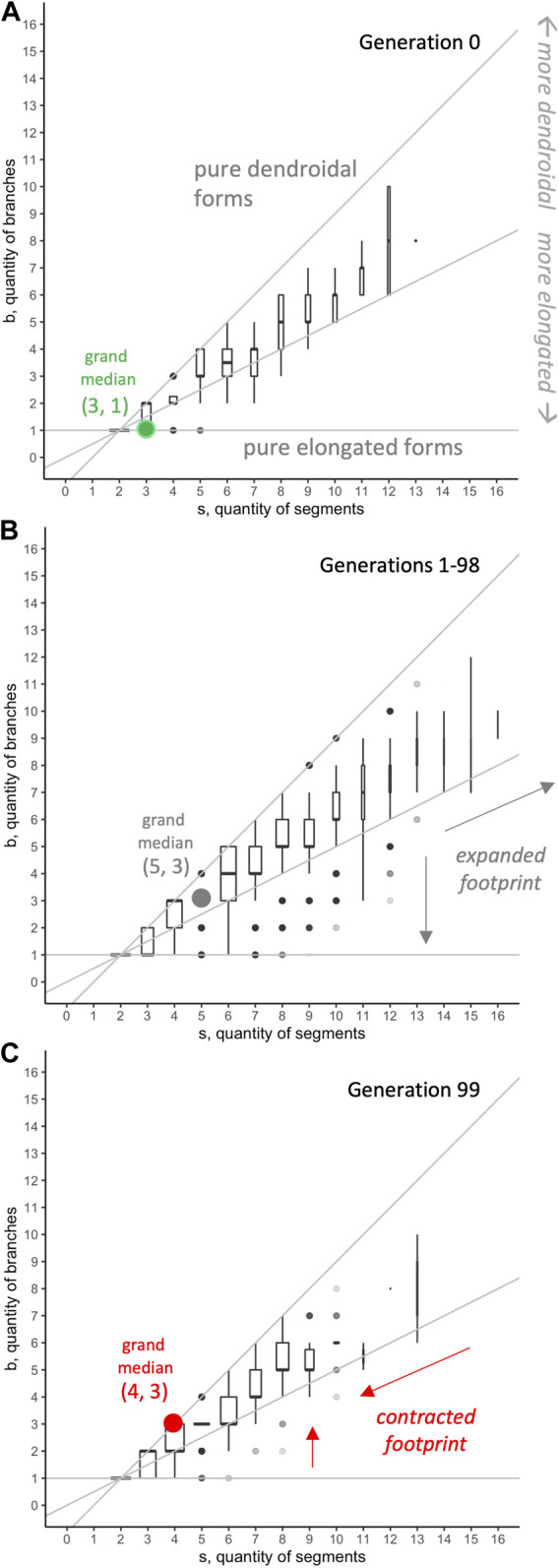
Morphospace analysis: distribution of s‐b body forms. In generation 0 **(A)**, the randomly generated morphologies show a clear dendroidal bias, with all median values falling in the region where body forms are more dendroidal than elongated. That same dendroidal bias is present in generations 1–98 **(B)**, even as selection expands the footprint of the distributions. The final generation, 99 **(C)**, has a dendroidally biased distribution with a contracted footprint relative to that of generation 0. The width of each boxplot is scaled to the square root of the number of observations in the group. All 594,000 individuals are represented.

Seeking to understand why only some positions in the s‐b morphospace have been occupied, we first examined the spatiotemporal distribution of forms (Figure 10). For the first generation (Figure 10A), which was randomly generated, the median number of individuals was unevenly distributed, falling above the $\frac{b}{s}=\frac{1}{2}$ line, indicating forms more dendroidal than elongated. Because this initial generation was not the result of selection, the distribution of forms represents development but not selection. When the populations were under selection, from generations 1 to 98 (Figure 10B), the footprint of the combined distributions expanded into larger values of s and b ; in spite of forms occurring below $\frac{b}{s}=\frac{1}{2}$ , the median value of b at each level of s indicates that dendroidal forms are more likely than elongated forms to evolve. Intriguingly, the distribution footprint of the final generation, 99 (Figure 10C), is contracted, with reduced magnitudes of s and b relative to the first and intermediate generations.

**FIGURE 11:**
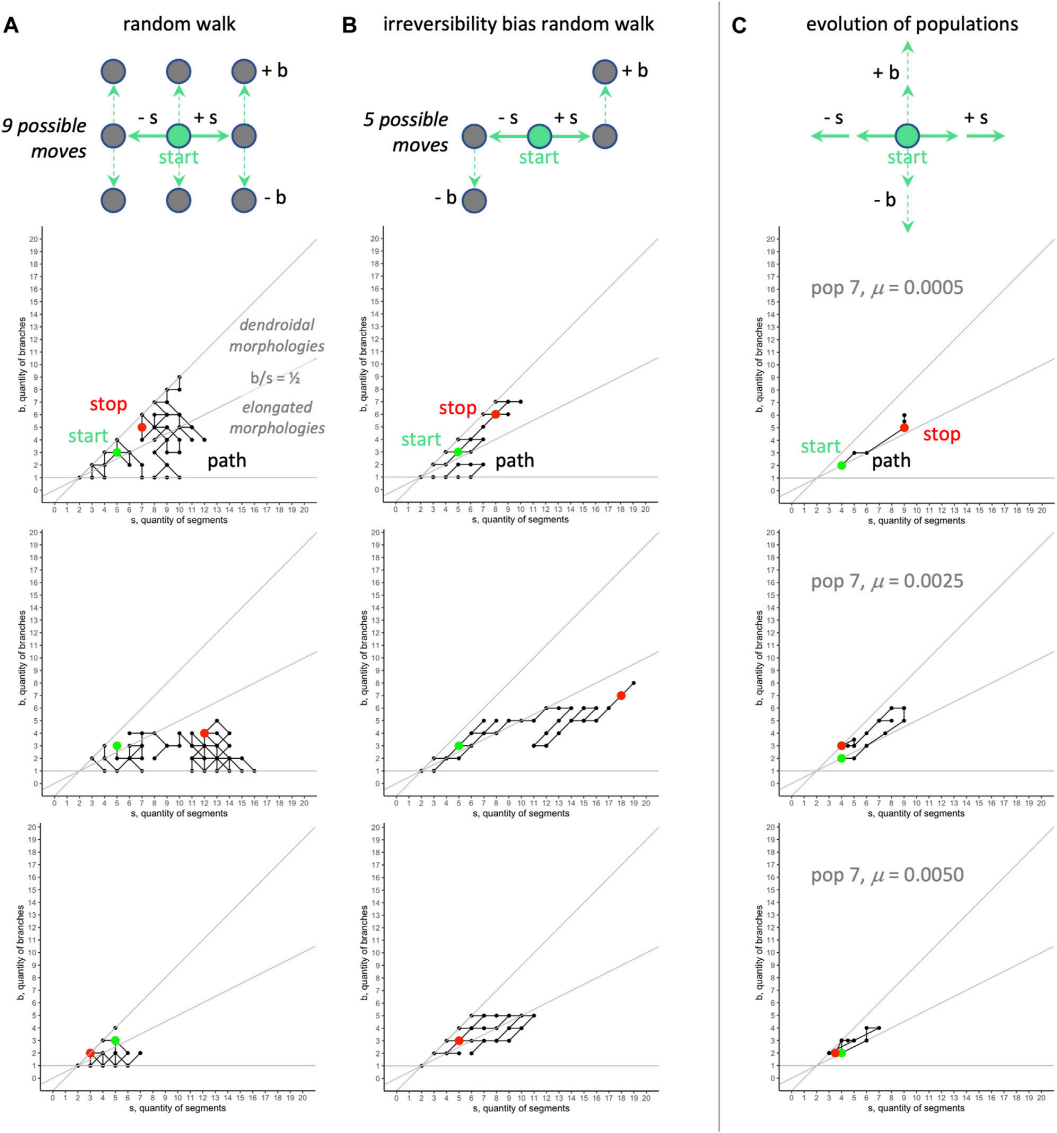
Random morphospace walk analysis: examples. Random walks **(A)** can move in any direction, and move into dendroidal and elongated sections of morphospace in these three examples. The irreversibility bias **(B)**, representing a possible developmental bias, causes the random walk to move diagonally with positive slope. For comparison, the evolutionary trajectories of three populations **(C)** tend to occur in the dendroidal morphologies section of the morphospace (for aggregate view, see Figure 10). The same random seed is used for the row-wise comparisons in the random walks. The random models start with the mean value of morphology evolved over all generations, s=5 and b=3 .

**FIGURE 12:**
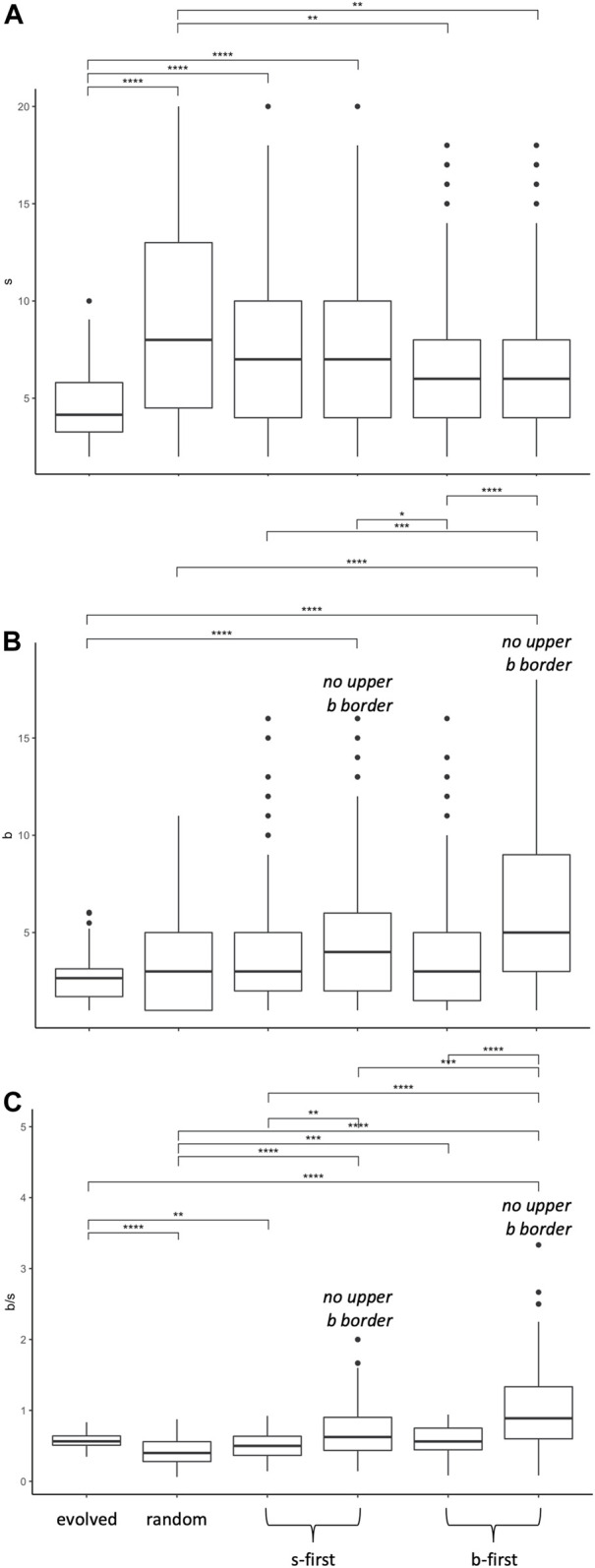
Morphospace walk analysis: evolution in s‐b morphospace is not a random walk. The final positions of the population (evolved, generation 100) and random walks (step 100) are compared in 99 different instances (99 evolutionary runs from 11 mutation levels and nine populations compared to 99 different random walks for each time of model). As shown by significant (*) Wilcoxon pairwise tests, the evolved populations differ from the random models in different ways depending on the metric. For segments s **(A)**, the evolved populations differ from the random walk and the s-first biased random walks. For branches b **(B)**, the evolved populations differ from the two biased random walks that lack an upper boundary for b . For the $\frac{b}{s}$ ratio **(C)**, the evolved populations differ from the random walk and the s -first biased walk that lacks an upper boundary for b .

When comparing various kinds of RMWs to our biorobot populations’ evolutionary walks in s‐b morphospace (see Figure 11 for some example walks), the movements of the evolved populations, as measured by their final positions, differ significantly from the five random models (Figure 12). For segments, the evolved populations differ from the random walk and the s -first biased random walks, with the models’ final positions in this dimension greater than that of the evolved populations. For branches, the evolved populations differ from the two biased random walks that lack an upper boundary. For the $\frac{b}{s}$ ratio, the evolved populations differ from the random walk and the s -first biased walk that lacks an upper boundary. In aggregate, the evolved populations are statistically distinguishable—in at least one of these three features—except for the model that is b -first with an upper boundary.

**FIGURE 13:**
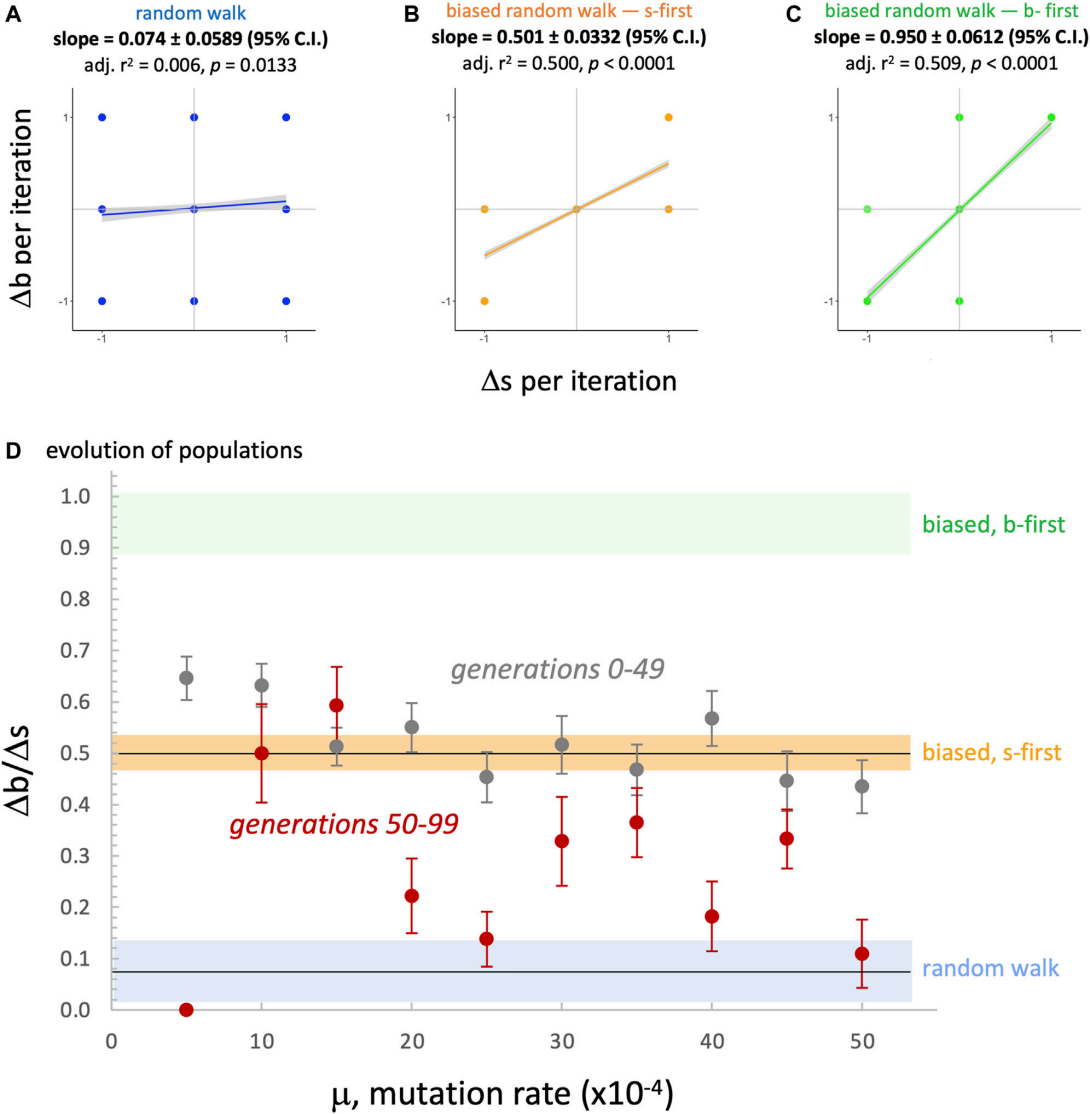
Random morphospace walk analysis: developmental bias in evolution? From the simplified set of steps available for a random walk and developmentally biased random walks, we expect three different relationships between the rate of change of b and s **(A–C)**. Data for these models is calculated from nine runs of each, with 99 generations (n=891) . In evolving populations **(D)**, evidence for a possible developmental bias in evolution is seen in the overlap of 95% confidence intervals (error bars) with the biased, s-first random walk model (orange bar, vertical range = 95% confidence interval). For most levels of mutation rate, the evolved populations overlap for the first 50 generations but not that last 50. For those later generations, three levels of mutation overlap with the random walk (blue bar, vertical range = 95% confidence interval).

Since the evolved populations have an upper boundary, as imposed by development, the three random walks with upper boundary were used to compare the *rate of change* of the morphospace walks (Figure 13). Each type of random walk is characterized by its rate of change $\frac{db}{ds}$ (Figures 13A–C). These slopes, and 95% confidence intervals, were calculated using linear regression, with data from nine runs of each model, with 99 steps (n = 891); because the evolved populations show dramatically different rates of change in b and s over generations 0–49 compared to generations 50–99 (see Figure 5), the rates of change $\frac{db}{ds}$ were likewise partitioned by generation and rate of mutation (Figure 13D). In our evolving populations, the calculated $\frac{db}{ds}$ values are statistically indistinguishable from that of the s -first random walk model—for most mutation rates, the evolved populations overlap for the first 50 generations but not the next 50. For those later generations, three levels of mutation overlap with the $\frac{db}{ds}$ value for the random walk. Note that the calculated $\frac{db}{ds}$ ratios for the evolving populations never overlap with the value from the b -first random walk.

**FIGURE 14:**
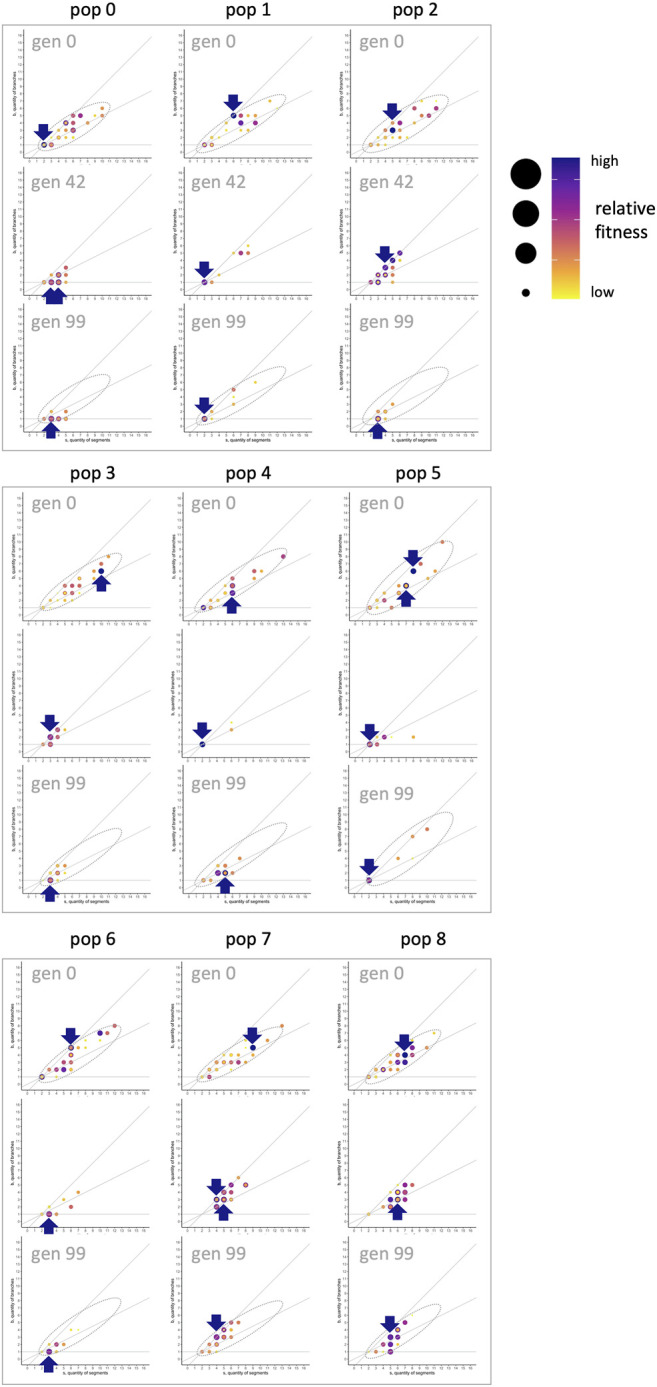
Morphospace and the shifting fitness landscape. To create fitness landscapes in the s−b morphospace, the relative fitness of the 60 individuals in a population is encoded by color ( μ fixed at 0.0025). In each of nine populations, the individual with the highest relative fitness (red point, red arrow) represents an adaptive peak at three different generations (0, 42, 99). Over generational time, note the shift in the fitness peak, showing that the adaptive landscape is in flux.

When morphospace is combined with evolutionary fitness, an adaptive landscape, also known as a fitness landscape, is created. For the evolved populations, tracking locations of individuals with the highest fitness, relative to others in that population and generation, shows that the fitness landscape changes over generational time (Figure 14). This is itself an important result, counter to intuitions that fitness landscapes are stable and absolute. In generation 0, when genomes are randomly generated, the highest fitnesses are seen in individuals with relatively high values of s and b . By generation 42, after the burst of evolutionary change characterized by a rapid increase in fitness (see Figure 4) and rapid change in morphology (see Figure 5), individuals with the highest fitness have shifted their relative positions in morphospace, now located predominantly in regions of low s and b . The similar positions of individuals with the highest fitness in the last generation indicates that the fitness landscape now is relatively stable.

## 6 Discussion

The evolution of robotic morphology is open-ended and complicated, but details can be exposed and investigated by aptly focused analytical methods: straightforward accounting illuminates the separate fates of different traits; selection gradient analysis uncovers how traits are targeted by selection and how that targeting varies over generational time; and morphospace walks can explore how randomness, development, and selection interact in morphological evolution. To demonstrate these methods, we evolved populations of bioinspired, embodied (segmented and branched) biorobots, which respond to selection on locomotion behavior by evolving their mechanical and sensorimotor morphologies. A straightforward accounting of fitness (Figure 4) and morphological forms (Figure 5) is an essential component of understanding the evolved morphologies, but it does not illuminate all of the relevant evolutionary driver processes. For example, it might be natural to think that selection is sufficient to explain morphological variance, but analyses in this paper have shown otherwise—the story of evolved morphology is more complex than that. That complexity is shown by the results of selection gradient analysis, which correlates fitness to morphology in a given generation and population at a given level of mutation.

Prior to running evolutionary trials, one might pick a morphological trait or functionally related set of traits as the most likely to affect fitness. In this exploratory work, we did not provide an *a priori* hypothesis, but our expectation was that selection would find and target the most important morphological traits; implied in that expectation is that the morphological targets would be the same across populations and over generational time. But that was not the case: While SG analysis revealed that morphology was always correlated with fitness, the specific morphologies varied by population and generation (Figure 6). A closer look at the patterns reveals that the mechanical morphological traits— s , b , $\frac{b}{s}$ , and average branch length—have the highest magnitude β coefficients. We take this to mean that these four mechanical traits are more important than the eight sensorimotor traits.

That claim is supported by a tally of how often mechanical and sensorimotor traits are selected in SG analysis (Figure 7). No matter the rate of mutation, a median of one mechanical and one sensorimotor trait are selected in each model (Figures 7A,B); by chance alone, we would expect a difference in the rate of occurrence to be proportional to $\frac{1}{3}$ (i.e., $\frac{4}{12}$ ) and $\frac{2}{3}$ (i.e., $\frac{8}{12}$ ), respectively. We note the differences in the direction of the inter-quartile range from the median, but the result that the central tendencies are the same is unexpected, suggesting that mechanical traits are over-represented in the models. We argue that selection is targeting the mechanical morphological system as an integrated whole, with different elements of that system rising to the level of statistical significance at different times.

The importance of mechanical morphology receives further support when we examine trait-by-trait occurrences (Figures 7C,D). At the lowest mutation rate, 0.0005, all mechanical traits occur more often than any sensorimotor trait; at higher mutation rates (0.0030 and higher), the $\frac{b}{s}$ ratio occurs between 30 and 40% of the time, well above the level of the sensorimotor trait with the highest values in that range (neurons, between 18 and 25%). Note that the $\frac{b}{s}$ ratio, by virtue of it being a ratio, is an integrated trait; that it becomes the most important of the mechanical traits at higher levels of mutation indicates, we believe, that the relation between b and s is integral to understanding the role of selection in these biorobots’ evolved morphologies.

Genetics and development create evolutionary mechanisms that operate alongside selection to alter morphology over generational time. The genetics of reproduction impinges, accumulating deleterious mutations such that some genomes fail catastrophically in every generation, reducing the mean fitness of the population, while also increasing the maximal fitness of the best individuals (Figure 4). Development grows the agents by elongation and branching, with an apparent bias in morphospace towards dendroidal forms (Figure 10). Thus, selecting for improved locomotion creates evolutionary responses that depend on multiple, co-occurring mechanisms.

The analytical approach in this paper reflects and illuminates a fundamental question, encompassing all relevant mechanisms, and applying here as it does to any embodied evolutionary system: What prevents all possible morphologies from occurring over generational time? Whether the goal is to understand the science and mechanisms that result in evolved morphology (e.g., when scientists work with biorobots) or the methods that lead to maximally fit individuals (as is common in evolutionary robotics), it is of interest to understand limits of morphological evolution. Due to a developmental algorithm in which elongation has precedence over branching—branching only occurs when elongation is no longer possible with available resources (Figure 2)—we expect more elongated than dendroidal forms. There are no properties that would prohibit, for example, a body with one branch and 10 segments (a missing combination in our populations, Figure 9), although the number of genes would limit the number of parts (segments, joints, etc.) at some undetermined level, presumably beyond the s and b limits of 16 and 12 observed in our populations. Thus, for the forms that do and don’t occur here, we hypothesize particular effects from evolutionary drivers such as randomness, development, and selection, resulting in these morphologies.

Our s‐b morphospace is unevenly occupied (Figure 9), with elongated body forms $\left(\frac{b}{s}<\frac{1}{2}\right)$ very rarely occurring at higher values of s (s>12) where dendroidal body forms $\left(\frac{b}{s}>\frac{1}{2}\right)$ occur. Looking at the timing and density of occurrences (Figure 10) adds complicating detail: The predominantly dendroidal distribution of the initial, randomly generated morphologies (generation 0) suggests the presence of a bias in the developmental algorithm. Under selection, from generations 1 to 98, the distribution expands to include more elongated forms; if a developmental bias towards dendroidal forms is in fact present, then selection overcomes this bias to the extent that elongated forms can be evolved. Continuing that interpretation, the final generation (99), with its contracted distributional footprint, is consistent with a developmental bias for dendroidal forms and selection acting on some or all of s , b , and the $\frac{b}{s}$ ratio.

We can state the above interpretation as the hypothesis that the position of the evolved populations in morphospace is caused primarily by selection and a developmental bias. Because the actual developmental process involves adding segments and never removing them during development (Figure 2), we call this an *irreversibility bias* (and the *s-first* model, in contrast to an alternative *b-first* model; see Section 4.4). We modeled this idea of irreversibility by noting that if development constrains evolutionary possibilities, then adding or removing a segment over generational time constrains the options for moving in morphospace (see Figure 11, top row, center); the corresponding random walk shows that movements are indeed biased, with the ratio $\frac{db}{ds}$ approximately equal to ½ (Figure 13). This biased s -first walk is built on top of a purely random walk (Figure 11), which produces a very small, but statistically non-zero, $\frac{db}{ds}$ value. Both the purely random and biased random walks differ from the placements (Figure 12) and rates of change (Figure 13) of the evolved populations. (Finding that selection for improved locomotion results in morphologies not expected by random chance is consistent with Auerbach and Bongard (2014), with different experimental conditions.) Further inspection, however, reveals the results are more complicated than that: The substantial overlap in the rates of change with the irreversibility bias precludes rejection of the hypothesis that development is an evolutionary driver.

In our biorobots, the development algorithm is made explicit and designed to enable study of such potential driver mechanisms, but even in such carefully conceived robot systems, interactions among intentionally designed and implemented components may produce unforeseen causes and effects. Indeed, the command of variables and values afforded by the computational-robotic paradigm enables more detailed explorations than could be achieved with living biological organisms, but because roboticists design the algorithms for their systems, they assume the extra responsibility for understanding potentially unintended effects of their implemented processes—including, but not limited to, unintended biases in development algorithms. One of our goals in presenting the analytical methods in this paper is to enable bioroboticists (and other scientists and roboticists) to analyze the effects of their designs from a basis of observed behavior and morphology, an alternative to approaches that intrinsically focus on encodings (e.g., Rothlauf, 2002; Miras, 2021); alternate perspectives can potentially illuminate unforeseen effects of design.

This applies directly to unforeseen effects of our biorobots’ design that arose in their evolutionary dynamics, stemming from development. As noted above, our development algorithm gives precedence to elongation (Figure 2), but the morphology of our robots prior to selection shows a tendency toward dendroidal forms (Figure 10A). We hypothesize that this is due to interacting factors of segmentation and branching in development, as illustrated in Figure 2. Segments are added in series until local resources in the newest segment are depleted; the algorithm then searches the body, looking for an open mount upon which to form a new branch. The lower a $\frac{b}{s}$ ratio is in the developing robot, the more segments are available for secondary branching: In our algorithm, terminal segments are the only ones that cannot be loci for new branches, because if resources permitted further extension from such a segment, it would lead to elongation; so, all other factors being equal, elongated forms have more opportunities to branch than dendroidal forms when a new segment is added. Thus, there exists a developmental bias towards branching that co-occurs with the bias of giving precedence to elongation, illustrating the intricacy of developmental biases that can exist in the complex developmental systems that lead to complex morphologies.

Modeling and exposing developmental bias invites us to consider its impact on evolution. In our studies, given that only the final adult forms (i.e., those that result from the completed developmental process and do not further change; cf. Kriegman et al., 2018) are tested and selected, we did not directly consider the effects of developmental bias on evolutionary response. But each adult in a population achieves its final form and place in the morphospace by developing to that position. If developmental bias were left out of the analysis of the evolution of morphology, selection might be assigned as the primary causal agent of the evolution of more dendroidal forms $\left(\frac{b}{s}>\frac{1}{2}\right)$ . But for our robot populations and traits analyzed, analysis of selection reveals that variation in adult morphology predicts only a fraction of variation in fitness (Figure 8). We draw the tentative conclusion that development acts as an evolutionary force alongside selection and mutation, and that a developmental irreversibility bias may have more impact in generations 0–49 than in generations 50–99 of our robots’ evolution.

Considering such support for the impact of developmental biases on evolved morphologies, the selection gradient analysis suggesting that selection on morphology accounts for only a portion of the variance in fitness (Figure 8), and that the trait $\frac{b}{s}$ involving branch-segment interactions seems to have particular importance in selection (Figure 7), our results suggest that an explanation of complicated evolved morphologies will be complicated, including interacting developmental biases, morphological traits, and evolutionary driver mechanisms. The selection gradient and morphospace walk methods employed for the above analyses enable illumination and comprehension of these interactions and complications, and they are inherently bioinspired, derived from an organismal view of biology that overlaps constructively with the perspectives needed for analyzing evolved robots. Moreover, because of their foundation in biology—which lacks the internal representational knowledge afforded by computational and robotic studies—the methods require only externally accessible observations of traits and behaviors. More generally, these bioinspired methods can be applied broadly to a range of driver mechanisms, fitness landscapes, and kinds of robots, and they can be illuminating additions to the analytical toolkit of roboticists seeking to understand their robots’ evolved morphologies.

## Data Availability

The raw data supporting the conclusion of this article will be made available by the authors, without undue reservation.
